# LncRNA LUCRC Regulates Colorectal Cancer Cell Growth and Tumorigenesis by Targeting Endoplasmic Reticulum Stress Response

**DOI:** 10.3389/fgene.2019.01409

**Published:** 2020-01-31

**Authors:** Guo-Hui Tang, Xue Chen, Jian-Cheng Ding, Jun Du, Xiao-Ting Lin, Lu Xia, Jia-Bian Lian, Feng Ye, Xiu-Sheng He, Wen Liu

**Affiliations:** ^1^Hunan Provincial Key Laboratory of Cancer Cellular and Molecular Pathology, University of South China, Hengyang, China; ^2^Cancer Research Institute of Hengyang Medical College, University of South China, Hengyang, China; ^3^Department of Anus and Bowels, Affiliated Nanhua Hospital, University of South China, Hengyang, China; ^4^Fujian Provincial Key Laboratory of Innovative Drug Target Research, School of Pharmaceutical Sciences, Xiamen University, Xiamen, China; ^5^Department of Medical Oncology, The First Affiliated Hospital of Xiamen University, Xiamen, China; ^6^State Key Laboratory of Cellular Stress Biology, School of Pharmaceutical Sciences, Xiamen University, Xiamen, China

**Keywords:** colorectal cancer, long non-coding RNA, unfolded protein response, cell growth, therapeutic target

## Abstract

Colorectal cancer (CRC) is the second most common cause of cancer-related death worldwide, and is well known for its strong invasiveness, rapid recurrence, and poor prognosis. Long non-coding RNAs (lncRNAs) have been shown to be involved in the development of various types of cancers, including colorectal cancer. Here, through transcriptomic analysis and functional screening, we reported that lncRNA LUCRC (LncRNA Upregulated in Colorectal Cancer) is highly expressed in colorectal tumor samples and is required for colorectal cancer cell proliferation, migration, and invasion in cultured cells and tumorigenesis in xenografts. LUCRC was found to regulate target gene expression of unfolded protein response (UPR) in endoplasmic reticulum (ER), such as BIP. The clinical significance of LUCRC is underscored by the specific presence of LUCRC in blood plasma of patients with colorectal cancers. These findings revealed a critical regulator of colorectal cancer development, which might serve as a therapeutic target in colorectal cancer.

## Introduction

Colorectal cancer (CRC) is one of the most common digestive tract malignancies. Based on the statistics from International Agency for Research on Cancer in 2018, the morbidity of colorectal cancer ranked the third and mortality the second among all cancer types (Bray et al., 2018). Substantial progresses have been made in early diagnosis and treatment of colorectal cancers, and the number of newly diagnosed cases was decreasing in recent years. However, there are still urgent needs for better ways of preventing and treating colorectal cancers due to its high morbidity and mortality (Recio-Boiles et al., 2019a; Recio-Boiles et al., 2019b).

Though the exact cause remains unclear, both environmental and genetic factors have been suggested to be tightly associated with colorectal cancer (Board, 2002). About 70% of patients with colorectal cancer have an average age of over 50 years old, and environmental factors were considered to play a major role in these patients. However, for patients under 50 years old, hereditary risk factors are the major cause. Around 5% of patients with colorectal cancer are attributed to two genetic diseases, Familial Adenomatous Polyposis (FAP) and Lynch syndrome. In addition, 10% of patients carry high-risk mutations (Peters, 2019). Colorectal cancer can be divided into five stages based on the extent of tumor invading the surrounding tissue and whether lymph node and distant metastasis occur. Colorectal cancers in stage 0, I, and II are without metastasis, and stage III are with local lymph node metastasis, while stage IV are with distant metastasis such as liver and lung. Surgical treatment is currently the most common treatment for colorectal cancer. In addition, there are treatments such as chemotherapy, radiotherapy, targeted therapy, and neo-adjuvant therapy (Brenner et al., 2014). Despite continuous improvement in treatment, the survival rate of patients with advanced colorectal cancer is still not optimistic. According to SEER (Surveillance, Epidemiology, and End Results Program) statistics, the 5-year survival rate of patients with stage IV colorectal cancer is only 5% (Recio-Boiles et al., 2019a; Recio-Boiles et al., 2019b). One of the main reasons for poor prognosis is the occultation of colorectal cancer and most patients are diagnosed at the advanced stage and died due to postoperative recurrence or metastasis. Therefore, finding more effective early diagnostic markers or therapeutic targets is of great significance for improving the early diagnosis rate and improving the survival rate of colorectal cancer (Reimers et al., 2013).

Protein-coding genes account for only 2% of the entire human genome, while the rest of the genome DNA was considered as “junk”. However, in the past decade, with the development of high-throughput sequencing technology, it has been found that a large number of transcripts are produced in these so called “junk regions”, and these transcripts are so called non-coding RNAs, which mainly include long non-coding RNAs (lncRNAs), microRNAs (miRNA), circular RNA (circRNA), enhancer RNA (eRNA), and others (De Santa et al., 2010; Kim et al., 2010; Djebali et al., 2012; Salzman et al., 2012). Among them, lncRNA has recently gained wide attention because of its important role in both physiological and pathological conditions. LncRNA is a kind of RNA molecule with a length greater than 200 nucleotides, which usually has no coding ability and performs biological functions in the form of RNA. However, with the advancement of proteomic mass spectrometry and the development of other new technologies, such as ribosome profiling, it has been shown that some lncRNAs can also encode microproteins, which are usually less than 100 amino acids in length and often play important functions such as embryogenesis (Pauli et al., 2014), muscle contraction and muscle regeneration (Anderson et al., 2015; Nelson et al., 2016; Matsumoto et al., 2017), immune response (Jackson et al., 2018), and tumor cell proliferation, migration, and invasion (Huang et al., 2017).

The biological function of lncRNA in cells depends mainly on their interaction with other biomolecules, including protein, DNA, and RNA. For lncRNA localized in the nucleus, they often participate in the transcriptional regulation of genes, either *in cis* or *in trans*, through binding to transcription factors or other factors (Kopp and Mendell, 2018). In addition, they can directly participate in the organization of the nuclear structure, thereby coordinating other processes in transcription, RNA processing, and gene expression (Fox and Lamond, 2010; Kopp and Mendell, 2018). For lncRNA localized to the cytoplasm, they can bind to RNA molecules such as mRNA or miRNA to affect post-transcriptional regulation processes such as protein translation or degradation. In addition, they can also regulate signal transduction by direct binding to proteins (Geisler and Coller, 2013; Lee et al., 2016; Wang et al., 2017; Zhang et al., 2018).

LncRNAs have been shown to play important regulatory roles in a variety of cellular processes including cell proliferation, differentiation, and development, and the aberrant expression of lncRNAs has been suggested to be involved in human diseases, such as cancer (Latos et al., 2012; Engreitz et al., 2013; Bouckenheimer et al., 2016; Renganathan and Felley-Bosco, 2017). In colorectal cancer, it has been reported that several lncRNAs can directly bind to proteins or act as miRNA sponges to participate in cellular signaling pathways such as β-catenin, p53, JAK/STAT, AKT/mTOR, and NF-κB, among others, thereby affecting cell cycle progression and/or EMT (epithelial mesenchymal transition) to control tumor cell growth, migration, and invasion (Kawasaki et al., 2016; Yang et al., 2017; Jiang et al., 2018; Wang et al., 2018; Sun et al., 2018; Tang et al., 2019). Many lncRNAs, such as CCAT1 and HOTAIR, are often associated with prognosis in clinical patients, and can be used as potential diagnostic markers for colorectal cancer (He et al., 2014; Svoboda et al., 2014). There are also lncRNAs, such as UCA1 and CCAL, that participate in tumor chemoresistance and radiosensitivity (Bian et al., 2016; Ma et al., 2016).

Endoplasmic reticulum (ER) homeostasis is critical for normal cellular activities. Under normal physiological conditions, BIP interacts with the stress signal receptor proteins IRE1, ATF6, and PERK located on the ER membrane, inactivating the three sensory proteins. When the cells are experiencing abnormal conditions, such as hypoxia, inflammation, and aberrant levels of calcium ion, ER stress is induced. ER stress will lead to the accumulation of misfolded proteins, which attract BIP and lead to the activation of the intracellular unfolded protein response (UPR) to promote protein folding and clearance of misfolded proteins (Wang and Kaufman, 2016). The molecular chaperone protein BIP (also known as GRP78 or HSPA5) itself is a major protective protein rapidly produced by ER stress and is recognized as one of the most sensitive marker proteins for ER stress, which in turn accelerates protein folding. Long-term UPR induces apoptosis *via* the PERK-eIF2α-ATF4-CHOP pathway, which activates apoptotic gene expression (Wang and Kaufman, 2016). Tumor cells grow faster and have a greater demand for nutrients. However, they have long been in a microenvironment such as hypoxia, acidosis, and nutrient deficiencies. Therefore, tumor cells can activate the UPR pathway to upregulate the expression of ER chaperone protein, promoting protein folding and clearance of misfolded protein to restore ER homeostasis and tolerate adverse effects of hypoxia, acidosis, and nutrient deficiencies. This mechanism is utilized by tumor cells to reduce cell apoptosis, promote tumor development and drug resistance, and even induce immune tolerance of tumor cells (Wang and Kaufman, 2014). Studies have shown that lncRNA in tumors can promote the activation of UPR pathway, such as lincRNA-p21, MEG3, and others (Yang et al., 2015; Zhang et al., 2019).

In summary, lncRNA plays an important regulatory role in the development of cancer, including colorectal cancer. However, the lncRNAs that are differentially expressed and functionally important for cell proliferation in colorectal cancer have not been systematically identified, and the molecular mechanisms remain unclear. Here, we systematically identified lncRNAs that are differentially expressed in colorectal tumor tissue and normal tissue samples by transcriptomic analysis. Further functional study revealed that lncRNA LUCRC (LncRNA Upregulated in Colorectal Cancer), among others, is important for the proliferation, migration, invasion, and tumorigenesis of colorectal cancer cells. Mechanistically, LUCRC was found to regulate the expression of UPR target genes, such as BIP. The clinical significance of LUCRC is underscored by the presence of LUCRC in blood plasma of patients with colorectal cancers.

## Materials and Methods

### Tissue and Blood Samples

**Figure 1:**
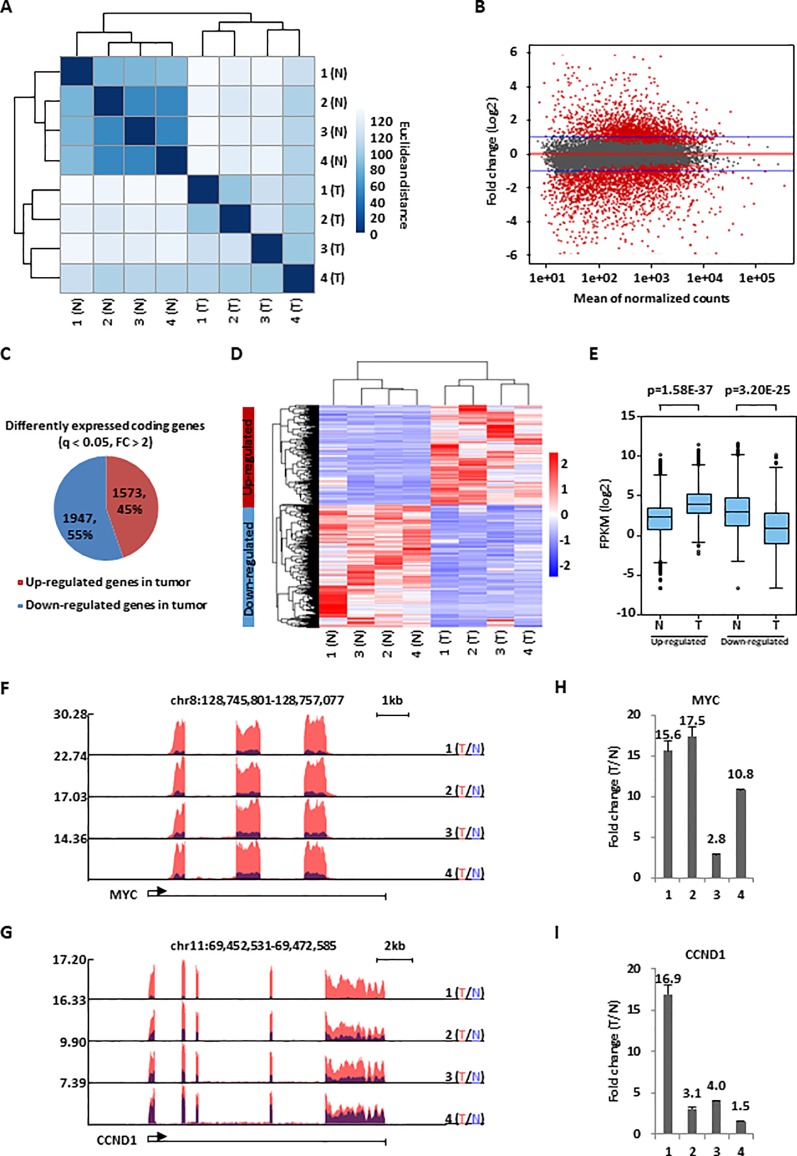
A large cohort of genes were dysregulated in colorectal cancer. **(A)** Colorectal tumor tissues (T) and the corresponding adjacent normal tissues (N) (n = 4 pairs) were collected and subjected to RNA-seq analysis followed by hierarchical cluster analysis. **(B)** MA plot shows the fold change (FC, tumor/normal, log2) against the average of normalized counts for all the samples as described in **(A)**. Red dots represented genes with significant change in tumor tissues (q <0.05), and blue line indicated fold change of two. **(C)** Pie chart shows genes dysregulated, both up-regulated and down-regulated, in colorectal tumor samples as described in **(A)** (q < 0.05, FC > 2). **(D, E)** Heat map **(D)** and box plot **(E)** representation of the expression levels (FPKM, log2) for genes, both up-regulated and down-regulated, in colorectal tumor samples as described in **(C)**. **(F, G)** UCSC genome browser views of RNA-seq as described in **(A)** for specific genes were shown as indicated. **(H, I)** RNA samples from four colorectal cancer patients as described in **(A)** were subjected to RT-qPCR analysis to examine the expression of MYC **(H)** and CCND1 **(I)**. Data was presented as fold change of tumor (T) versus normal (N) as indicated (± s.e.m.).

Colorectal tumor tissues and matched adjacent normal tissues were collected during tumorectomy after receiving permission from patients. All tissue specimens were immediately frozen in liquid nitrogen and stored at −80°C until RNA extraction. Peripheral whole-blood samples from colorectal cancer patients and healthy controls were collected in EDTA anticoagulation tubes, immediately centrifuged at 3,000 rpm for 8 min to separate plasma and stored at −80°C until RNA extraction. The diagnosis of colorectal cancer was histopathologically confirmed. The stage classification of tumor samples used in this study was listed as following. To identify genes that are dysregulated in colorectal cancer, four pairs of tumor and the adjacent normal tissues were collected from patients with either stage II (n = 1) or stage III (n = 3) colorectal cancer (**Figure 1**); To validate the expression of LUCRC in colorectal cancer, fourteen tumor and the adjacent normal tissues were collected from patients with either stage III (n = 12) or II (n = 2) colorectal cancer (**Figures 3K** and **4K**); To examine the expression of LUCRC in blood, blood samples were collected from seven patients with either stage III (n = 3) or IV (n = 4) colorectal cancer (**Figure 5**). The study was approved by the Institutional Ethics Committee of Affiliated Nanhua Hospital, University of South China and The First Affiliated Hospital of Xiamen University. All research was performed in compliance with government policies and the Helsinki Declaration. Experiments were undertaken with the understanding and written consent of each subject.

### Molecular Cloning

ShRNAs (small hairpin RNAs) targeting LUCRC were cloned into lenti-viral pLKO.1 vector between Age and EcoRI restriction enzyme sites (targeting sequence: GCTGCTAGAGAAGAGGTACAG (shLUCRC-1); GTCAGCCCATCACCGAAAGAA (shLUCRC-2)).

### RNA Isolation and RT-qPCR

Total RNA was isolated using Trizol (Takara) following the manufacturer’s protocol. First-strand cDNA synthesis from total RNA was carried out using All-In-One RT MasterMix (Abm), followed by quantitative PCR (qPCR) using AriaMx Real-Time (RT) PCR machine (Agilent Technologies). RNA samples from three biological repeats were pooled together for RT-qPCR analysis, and at least three technical repeats have been done for each pooled sample. Standard error of the mean is depicted. Sequence information for all primers used to check gene expression was presented in **Supplementary Table 1**.

### Immunoblotting Analysis

Immunoblotting analysis was performed as described previously (Gao et al., 2018). Anti-ATF6 (24169-1-AP) and anti-ACTIN antibodies were purchased from Proteintech, Inc.

### SiRNA and Plasmids Transfection, Lenti-Viral Vectors Packaging, and Infection

SiRNAs specifically targeting lncRNAs were purchased form RiboBio, and sequence information for all siRNAs were presented in **Supplementary Table 2**. SiRNA transfections were performed using Lipofectamine 2000 (Invitrogen) according to the manufacturer’s protocol. Plasmid transfections were performed using Polyethyleneimine (PEI, Polysciences) according to the manufacturer’s protocol. For lenti-viral vectors packaging and infection, HEK293T cells were transfected with lenti-viral vectors together with packaging vectors, pMDL, VSVG, and REV, at a ratio of 10:5:3:2 using Polyethyleneimine (PEI, Polysciences) for 48 h according to the manufacturer’s protocol. Virus was collected, filtered, and added to HCT116 cells in the presence of 10 μg/mL polybrene (Sigma, H9268).

### Cell Proliferation Assay, FACS (Fluorescence-Activated Cell Sorting) Analysis, Colony Formation Assay, Wound Healing Assay, Trans-Well Assay, and Xenograft Assay

Cell viability was measured by using a CellTiter 96 AQueous one solution cell proliferation assay kit (Promega) following the manufacturer’s protocol. Briefly, HCT116 cells were transfected with control siRNA (siCTL) or siRNA specifically targeting lncRNA, and maintained in RPMI 1640 medium followed by cell proliferation assay. Similarly, for lenti-virus infection, cells were infected with virus for 48 h and maintained in RPMI 1640 medium followed by cell proliferation assay. To measure cell viability, 20 μl of CellTiter 96 AQueous one solution reagent was added per 100 μl of culture medium, and the culture plates were incubated for 1–2 h at 37 °C in a humidified, 5% CO_2_ atmosphere incubator. Data was recorded at wavelength 490 nm using a Thermo Multiskan MK3 Microplate Reader.

For FACS analysis, cells were trypsinized, washed with PBS, and fixed with ethanol at −20 °C overnight. Cells were then washed with PBS and stained with PI/Triton X-100 staining solution (0.1% (v/v) Triton X-100, 0.2 mg/mL DNase-free RNase A (Sigma), 0.02 mg/mL propidium iodide (Roche)) at 37 °C for 15 min. DNA content was then measured and about 10^4^ events were analyzed for each sample. Data were analyzed using ModFit LT (Verity Software House).

For colony formation assay, around 2,000 cells were infected with control shRNA (shCTL) or shRNA specifically targeting LUCRC (shLUCRC) and maintained in a 6-well plate. Colonies were examined 10 days after. Briefly, colonies were fixed with methanol/acetic acid solution (3:1) for 5 min and stained with 0.1% crystal violet for 15 min. After washing with PBS extensively, colonies were photographed.

For wound-healing assay, cells transfected with siCTL or siLUCRC were re-seeded at confluence in 6-well plates, and wounds were created with a P200 pipette tip. After removing cellular debris by washing cells with PBS, three images of each well were taken. The wounded area was measured by using image J and recorded as A0. The cells were then allowed to migrate back into the wounded area, and images were taken to measure the wounded area 12, 24, and 48 h later and recorded as A1, A2, and A3, respectively. Cell migration was presented as wound closure (%) = (wounded area (A0-A1 or A2 or A3)/wounded area A0) × 100%.

For trans-well assay, cells transfected with siCTL or siLUCRC were re-seeded on the top compartment of transwell Boyden chambers (8 μm, Corning, USA) in serum-free media, and then allowed to migrate to the lower compartment contained complete media with 10% FBS (fetal bovine serum) in a humidified, 5% CO_2_ atmosphere incubator at 37°C. After 24 h, the inserts were washed with PBS and fixed with 4% paraformaldehyde for 15 min at 4°C and stained with 0.1% crystal violet for 10 min. After washing with PBS extensively, cells that did not migrate into the lower compartment were wiped away with a cotton swab and migrated cells were photographed.

For xenograft assay, two groups (5 mice/group) of female BALB/C nude mice (age 4–6 weeks) were subcutaneously implanted with 1 × 10^7^ of shCTL or shLUCRC cells suspended in PBS. All mice were euthanized 20 days after subcutaneous injection. Tumors were then excised, photographed, and weighted. Animals were housed in the Animal Facility at Xiamen University under pathogen-free conditions, following the protocol approved by the Xiamen Animal Care and Use Committee.

### RNA Sequencing (RNA-Seq) and Computational Analysis of RNA-Seq Data

Two biological repeats were subjected to RNA extraction and sample preparation. Total RNA was isolated using Trizol (Takara) following the manufacturer’s protocol followed by DNase I digestion to remove residual DNA. RNA library preparation was performed by using NEBNext^®^ Ultra™ Directional RNA Library Prep Kit for Illumina (E7420L). Paired-end sequencing was performed with Illumina HiSeq 3000 platform. Sequencing reads were aligned to the human reference genome (hg19) by using STAR (Dobin et al., 2012). Cufflinks was used to calculate the expression of RefSeq annotated genes with the option -M (reads aligned to repetitive regions were masked) and -u (multiple aligned read are corrected using ‘rescue method’) (Trapnell et al., 2012). Genes with FPKM (fragments per kilobase per million mapped reads) larger than 0.5 in either normal or tumor, or either siCTL or siLUCRC sample were included in our analysis. FPKM of a gene was calculated as mapped reads on exons divided by exonic length and the total number of mapped reads. DESeq2 was used to determine differentially expressed genes (Love et al., 2014). For differentially expressed genes in CRC clinical samples and siLUCRC-transfected samples, a cutoff of q value less than 0.05 and fold change larger than 2 (for clinical samples) or 1.5 (siLUCRC-transfected samples) was applied. Box plot and heat map were generated by R software and significance was determined using Student’s t-test. Gene Ontology (GO) and Kyoto Encyclopedia of Genes and Genomes (KEGG) pathway analysis were performed using Metascape (Zhou et al., 2019).

RNA-seq data were deposited in the Gene Expression Omnibus database under accession GSE136040. The following link has been created to allow review of record GSE136040 while it remains in private status: https://www.ncbi.nlm.nih.gov/geo/query/acc.cgi?acc=GSE136040 (token: edqvkikgjlsjdwn).

### Mining of the Cancer Genome Atlas (TCGA) Data

Expression data of lncRNAs in a cohort of colorectal tumor samples from TCGA was downloaded from GDC Data Portal. Box plots were generated by R software and significance was determined using DESeq2 package.

## Results

### Transcriptomic Analysis Revealed That a Large Cohort of Genes Were Dysregulated in Colorectal Cancer

To identify genes that are dysregulated in colorectal cancer, RNA-seq analysis was performed for colorectal tumor tissues and the corresponding adjacent normal tissues collected from four patients with colorectal cancer. Hierarchical cluster analysis indicated that the four tumor samples could be well distinguished from the four normal ones (**Figure 1A**). Differential gene expression analysis revealed that a large cohort of genes were significantly dysregulated in tumor samples. Specifically, 1,573 and 1,947 genes were found to be up- and down-regulated, respectively, in tumor samples compared to adjacent normal ones (q < 0.05, FC > 2) (**Figures 1B, C**). The expression of these genes were demonstrated by heat map and box plot (**Figures 1D, E**). Genes known to be dysregulated in colorectal cancers, such as MYC and CCND1 (He et al., 2014), were also found to be highly and significantly up-regulated in our RNA-seq analysis, which was further validated by RT-qPCR analysis (**Figures 1F–I**). Gene ontology (GO) and Kyoto Encyclopedia of Genes and Genomes (KEGG) pathway analysis indicated that genes with implications in ribosome biogenesis, cell cycle, DNA replication, immuno-response, and others were dysregulated in tumor samples (**Figures S1A–D**). Many of these genes were downstream targets of transcription factors known to be involved in colorectal cancer development, such as E2F1, MYC, and P53, suggesting our RNA-seq analysis is valid (**Figures S1E, F**).

### Transcriptomic Analysis Revealed That Hundreds of lncRNAs Were Dysregulated in Colorectal Cancer

**Figure 2:**
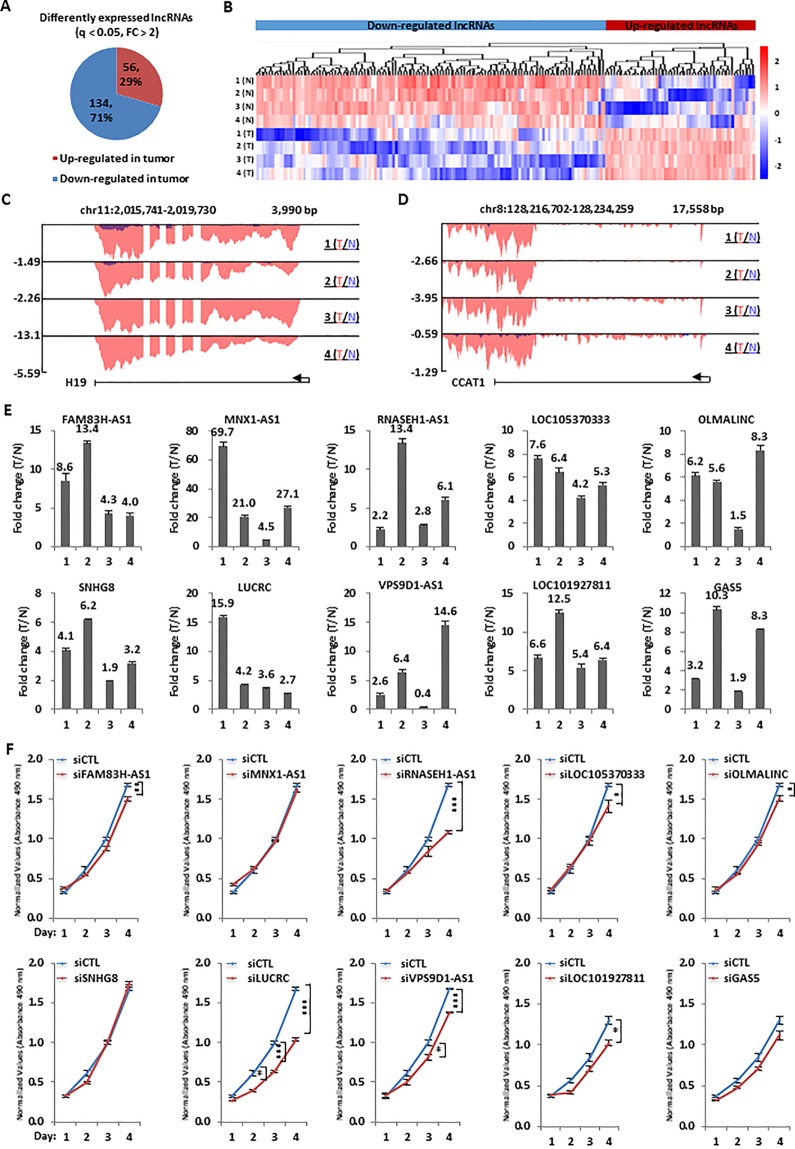
Hundreds of lncRNAs were dysregulated and a number of them were required for colorectal cancer cell growth. **(A)** Pie chart shows lncRNAs dysregulated, both up-regulated and down-regulated, in colorectal tumor samples as described in Figure 1A (q < 0.05, FC > 2). **(B)** Heat map representation of the expression levels (FPKM, log2) for dysregulated lncRNAs, both up-regulated and down-regulated, in colorectal tumor samples as described in **(A)**. **(C, D)** UCSC genome browser views of RNA-seq as described in Figure 1A for specific up-regulated lncRNAs were shown as indicated. **(E)** RNA samples from four patients with colorectal cancer as described in Figure 1A were subjected to RT-qPCR analysis to examine the expression of 10 top-upregulated lncRNAs in colorectal tumor samples as detected by RNA-seq analysis. Data was presented as fold change of tumor (T) versus normal (N) as indicated (± s.e.m.). **(F)** HCT116 cells were transfected with control siRNA (siCTL) or siRNA specifically targeting each individual lncRNA for duration as indicated followed by cell proliferation assay. (± s.e.m., *P < 0.05, **P < 0.01, ***P < 0.001).

We next analyzed our RNA-seq data above to identify the dysregulated lncRNA program in colorectal tumor tissue samples. It was found that the expression of 190 lncRNAs had at least two fold change compared tumor tissue samples to adjacent normal tissue samples, with 56 and 134 lncRNAs being up-regulated and down-regulated, respectively (**Figure 2A**). The expression of these 190 lncRNAs in both tumor and normal samples was shown by heat map (**Figure 2B**). LncRNA known to be up-regulated in colorectal cancer, such as H19 and CCAT1, were confirmed in our RNA-seq analysis. The UCSC genome browser views of H19 and CCAT1 were shown (**Figures 2C, D**). Similarly, representative lncRNAs that were found to be down-regulated in our RNA-seq analysis were also demonstrated (**Figures S2A–D**). In the current study, we focused on studying the function and molecular mechanism of the top up-regulated lncRNAs in colorectal cancers. Firstly, the expression of 10 top up-regulated lncRNAs was validated by RT-qPCR analysis (**Figure 2E**). The clinical relevance of these lncRNAs, except LOC105370333, was underscored by their significantly higher expression in a cohort of colorectal tumor tissues compared to adjacent normal tissues from TCGA (**Figure S2E**). We then tested whether these lncRNAs affect colorectal cancer cell proliferation by using HCT116 as a model cell line. HCT116 cells were transfected with control siRNA (siCTL) or siRNA specifically targeting each individual lncRNA, followed by cell proliferation assay. Out of the 10 lncRNAs being tested, knockdown of several of them resulted in growth defects of HCT116 cells and lncRNA LOC105371049 seemed to affect HCT116 cell growth the most (**Figure 2F**). The knockdown efficiency of siRNAs targeting the 10 lncRNAs was examined by RT-qPCR analysis (**Figure S2F**). We named lncRNA LOC105371049 as LUCRC (LncRNA Upregulated in Colorectal Cancer).

### LUCRC Was Required for Colorectal Cancer Cell Proliferation, Migration, and Invasion *In Vitro* and Tumorigenesis *In Vivo*

LUCRC is located on chromosome 16 of the human genome (chr16:2,141,437-2,145,018), and it is transcribed and spliced to contain three exons (600 bp in length) (**Figures S3A, B**). Polysome profiling revealed that LUCRC was mainly in fractions where free RNAs (unbound RNAs) found to be, suggesting that LUCRC is predominantly existed as a non-coding RNA (**Figure S3C**). As expected, ACTIN, a coding gene, was found to be predominantly associated with polysome fractions (**Figure S3D**). LUCRC was found to be mainly localized in the cytosol of cells as detected by cellular fractionation followed by RT-qPCR analysis (**Figure S3E**). ACTIN and MALAT1 mRNA were found to be mainly localized in the cytosol and nucleus of the cell, respectively, indicating our cellular fractionation was successful (**Figure S3E**).

**Figure 3:**
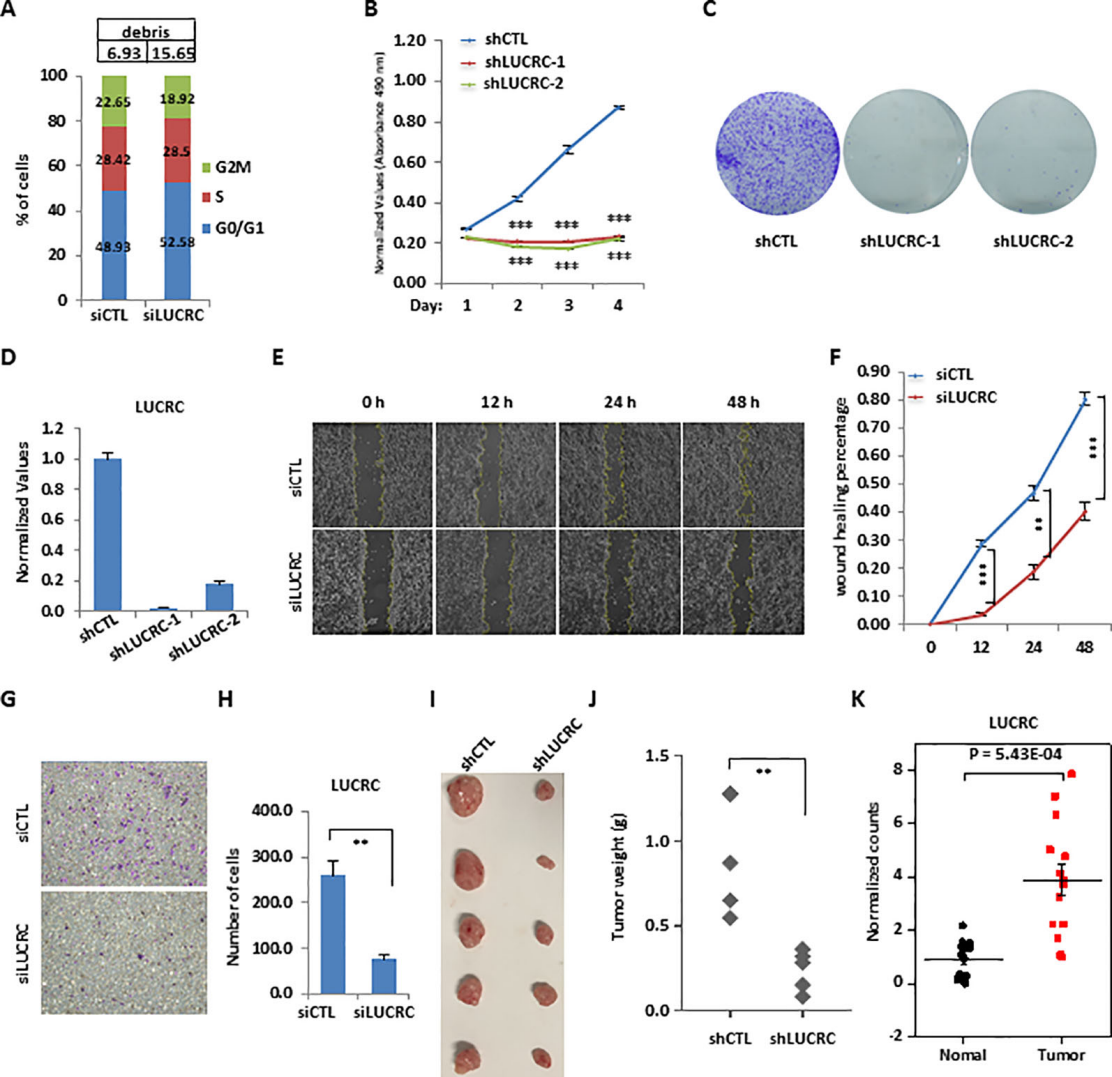
LUCRC was required for colorectal cancer cell proliferation, migration, and invasion *in vitro* and tumorigenesis *in vivo*. **(A)** HCT116 cells were transfected with control siRNA (siCTL) or siRNA specifically against (siLUCRC) for 3 days before FACS analysis. Percentage of apoptotic cells (debris) were also shown on the top. **(B)** HCT116 cells were infected with control shRNA (shCTL) or two independent shRNA specifically targeting LUCRC (shLUCRC) for duration as indicated before cell proliferation assay (± s.e.m., ***P < 0.001). **(C)** HCT116 cells were infected with shCTL or two independent shLUCRC for 10 days before colony formation assay (± s.e.m., **P < 0.01, ***P < 0.001). **(D)** HCT116 cells as described in **(B, C)** were subjected to RNA extraction and RT-qPCR analysis to examined the expression of LUCRC (± s.e.m., ***P < 0.001). **(E)** HCT116 cells were transfected with siCTL or siLUCRC for 2 days and then re-seeded at full confluence and maintained for duration as indicated before wound-healing assay. **(F)** Quantification of wound closure as shown in **(E)** (± s.e.m., **P < 0.01, ***P < 0.001). **(G)** HCT116 cells were transfected with siCTL or siLUCRC for 2 days and then re-seeded at full confluence and maintained for 1 day before transwell assay. **(H)** Quantification of cells as shown in **(G)** (± s.e.m., **P < 0.01). **(I)** ShCTL or shLUCRC-infected HCT116 cells were injected subcutaneously into female BALB/C nude mice for xenograft experiments. **(J)** Tumor weight as shown in **(I)** (± s.e.m., **P < 0.01). **(K)** Colorectal tumor tissues and the corresponding adjacent normal tissues were collected from a group of colorectal cancer patients (n = 14) and subjected to RNA extraction and RT-qPCR analysis to examine the expression of LUCRC (± s.e.m., ***P < 0.001).

To further characterize LUCRC regulation of cell growth, FACS analysis in HCT116 cells revealed that knockdown of LUCRC led to a significant increase of number of cells in G1 phase, the accumulation of which eventually led to cell apoptosis (debris) (**Figure 3A**). The critical role of LUCRC in HCT116 cell proliferation was independently confirmed by using two lenti-viral shRNAs targeting LUCRC (**Figure 3B**). Furthermore, HCT116 cells infected with shRNA targeting LUCRC exhibited much fewer colonies in colony formation assay (**Figure 3C**). The knockdown efficiency of shRNA targeting LUCRC was shown by RT-qPCR analysis (**Figure 3D**). LUCRC was also found to be required for the migration and invasion of HCT116 cells as examined by wound healing assay and transwell assay (**Figures 3E–H**). To test whether LUCRC is critical for tumorigenesis *in vivo*, nude mice was injected subcutaneously with control shRNA (shCTL) or shRNA targeting LUCRC (shLUCRC)-infected HCT116 cells. It was found that knockdown of LUCRC significantly impaired tumorigenesis in mice (**Figures 3I, J**). To further strengthen the role of LUCRC in colorectal cancer cell growth, we knocked down LUCRC in several other colorectal cancer cell lines including RKO and DLD1, and measured cell proliferation rate. It was found that, similar as what we observed in HCT116 cells, knockdown of LUCRC resulted in much slower proliferation rate in these cell lines tested (**Figures S3F, G**). The clinical relevance was underscored by the higher expression of LUCRC in a cohort of colorectal tumor tissues in house (n = 14) as well as those in TCGA compared to normal tissues (**Figures 3K** and **S2E**). It should be noted that the expression of LUCRC in colorectal cancers exhibited no significant difference among different tumor stages, and had no significant correlation with patient disease outcome (**Figures S3H, I**). Taken together, LUCRC is critical for the growth, migration, and invasion of colorectal cancer cells *in vitro* and tumorigenesis *in vivo*, and it is significantly up-regulated in colorectal tumor tissues.

### LUCRC Was Required for the Expression of Genes Involved in Endoplasmic Reticulum (ER) Stress Response, Including BIP

**Figure 4:**
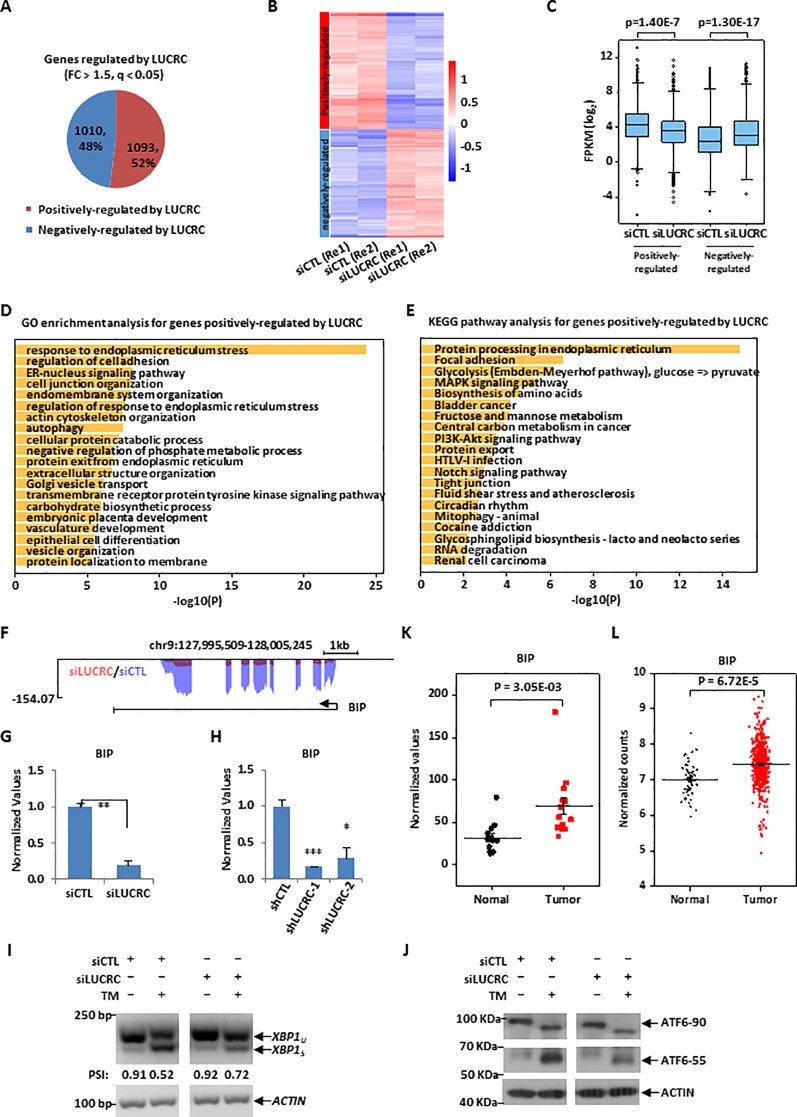
LUCRC was required for the expression of genes involved in ER stress response, including BIP. **(A)** HCT116 cells transfected with control siRNA (siCTL) and siRNA specifically targeting LUCRC (siLUCRC) for 3 days were subjected to RNA-seq analysis, and genes positively- and negatively-regulated by LUCRC were shown by pie chart (Fold change (FC) > 1.5). **(B, C)** Heat map **(B)** and box plot **(C)** representation of the expression levels (FPKM, log2) for genes positively- and negatively-regulated by LUCRC in HCT116 cells as shown in **(A)**. Re1: replicate 1; Re2: replicate 2. **(D, E)** GO **(D)** and KEGG **(E)** analysis for genes positively-regulated by LUCRC in HCT116 cells as shown in **(A)**. **(F)** UCSC genome browser view of RNA-seq as described in **(A)** for BIP was shown. **(G)** HCT116 cells were transfected with siCTL or siLUCRC for 3 days followed by RT-qPCR analysis to examine the expression of BIP (± s.e.m., **P < 0.01). **(H)** HCT116 cells were infected with shCTL or shLUCRC for 3 days followed by RT-qPCR analysis to examine the expression of BIP (± s.e.m., *P < 0.05, ***P < 0.001). **(I)** HCT116 cells were transfected with siCTL or siLUCRC for 3 days and then treated with or without tunicamycin (TM) (1μg/mL) for 8 h, followed by RNA extraction, reverse transcription and PCR analysis using primers targeting *XBP1* or *ACTIN*. Splicing of XBP1 was presented as PSI (percentage of inclusion: XBP1u/(XBP1u + XBPIs)). XBP1u: unspliced XBP1; XBP1s: spliced XBP1. DNA fragment size was indicated on the left. bp: base pair. **(J)** HCT116 cells as described in **(I)** were subjected to immunoblotting analysis by using antibodies as indicated. Molecular weight was indicated on the left. KDa: kilodalton. ATF6-90: full length ATF6; ATF6-55: partial ATF6. **(K)** Colorectal tumor tissues and the corresponding adjacent normal tissues were collected from a group of colorectal cancer patients (n = 14) and subjected to RNA extraction and RT-qPCR analysis to examine the expression of BIP (± s.e.m., **P < 0.01). **(L)** The expression of BIP in a cohort of clinical colorectal tumor (n = 647) and normal (n = 51) samples from TCGA (The Cancer Genome Atlas).

We next sought to identify the target genes regulated by LUCRC in order to gain insights into the signaling pathways it is involved in. To this end, HCT116 cells were transfected with control siRNA (siCTL) or siRNA specifically targeting LUCRC (siLUCRC) followed by RNA-seq analysis. The impact of LUCRC on gene expression between two biological repeats was highly correlated (**Figure S4A**). It was found that there were 1,093 and 1,010 genes positively- and negatively-regulated by LUCRC, respectively (Fold change > 1.5, q < 0.05) (**Figures 4A** and **S4B** and **Supplementary Table 3**). The expression of these LUCRC-regulated genes was shown by heat map and box plot (**Figures 4B, C**). To understand the biological functions and signaling pathways that LUCRC is involved in, GO and KEGG analysis was performed for genes regulated by LUCRC. As expected, multiple signaling pathways that are well known to be involved in cancer development were found to be present, with endoplasmic reticulum (ER) stress response being the most prevalent and significant (**Figures 4D, E**). ER stress response is induced by the increase of unfolded proteins in the ER of the cell, which attract the molecular chaperon BIP, resulting in the release of the three signal receptor proteins ATF6, PERK, and IRE1α on the ER membrane to activate the unfolded protein response (UPR) pathway. The downstream target genes of UPR pathway are mainly function in accelerating the correct folding of unfolded proteins and the removal of misfolded proteins. Among these target genes, the molecular chaperon BIP is one of the most important one to help protein folding. Indeed, BIP expression was found to be dependent on LUCRC in our RNA-seq analysis (**Figure 4F**), which was further confirmed by RT-qPCR analysis in cells transfected with siRNA or infected with shRNA specifically targeting LUCRC (**Figures 4G, H**).

We next sought to examine whether LUCRC is involved in UPR pathway to regulate BIP expression, HCT116 cells were transfected with control siRNA or siRNA specifically targeting LUCRC for 3 days and then treated with tunicamycin (TM), which were then subjected to analysis of some of the critical cellular events during the activation of UPR. It was found that TM treatment induced the splicing of XBP1 from XBP1u (unspliced) to XBP1s (spliced), an indicator of UPR activation, which was significantly attenuated when LUCRC was knocked down (**Figure 4I**). Similarly, knockdown of LUCRC led to a decrease of TM-induced processing of full length ATF6 (ATF6-90) to partial ATF6 (ATF6-55) (**Figure 4J**). However, the activation of PERK-eIF2α-ATF4 pathway was unaltered in response to LUCRC knockdown as indicated by eIF2α phosphorylation (data not shown). To underscore the significance of LUCRC regulation of BIP expression in tumorigenesis, BIP was found to be expressed significantly higher in a cohort of colorectal tumor tissues compared to adjacent normal tissues (**Figure 4K**), which was confirmed by TCGA datasets (**Figure 4L**). It should be noted that, similar as LUCRC, the expression of BIP in colorectal cancers exhibited no significant difference among different tumor stages, and had no significant correlation with patient disease outcome (**Figures S4C, D**). Taken together, our RNA-seq analysis revealed that LUCRC is involved in the regulation of UPR pathway, inducing the expression of BIP.

### LUCRC Was Detected in Blood Samples of Colorectal Cancer Patients

**Figure 5:**
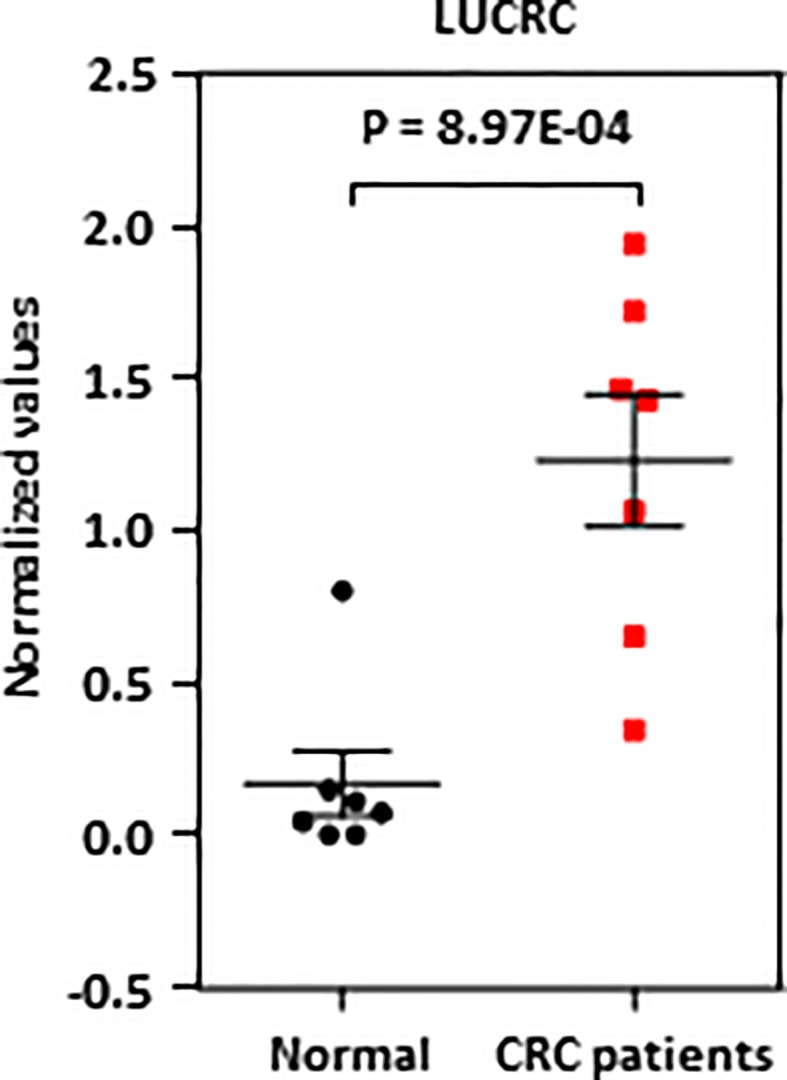
LUCRC was detectable in blood samples of colorectal cancer patients. Blood samples from seven colorectal cancer patients as well as seven healthy controls were subjected to RNA extraction and RT-qPCR analysis.

The high expression of LUCRC in colorectal tumor tissues and its requirement for colorectal cancer cell growth and tumorigenesis prompted us to examine whether it is differentially present in blood of colorectal cancer patients and healthy controls and therefore might serve as a diagnosis marker. To this end, blood samples from a group of colorectal cancer patients as well as healthy controls were subjected to RNA extraction and RT-qPCR analysis. It was found that LUCRC was nearly undetectable in six out of seven healthy control donors, but it was expressed in a significantly higher level in all seven colorectal cancer patients (**Figure 5**).

## Discussion

LncRNAs have been well known to be involved in the development of cancer, including colorectal cancer. Previous studies have demonstrated that lncRNAs that are dysregulated in colorectal cancer might serve as diagnosis makers and/or therapeutic targets (He et al., 2014; Yang et al., 2017; Wang et al., 2018). Through transcriptomic analysis of colorectal tumor and adjacent normal tissue samples, we systematically mapped the lncRNA program that is dysregulated in colorectal cancer. Besides the ones that were reported to be dysregulated in colorectal tumor samples, such as CCAT1, H19, DANCR, ZFAS1, SNHG16, CYTOR (He et al., 2014; Christensen et al., 2016; Yang et al., 2017; Fang et al., 2017; Wang et al., 2018; Wang et al., 2018), we have identified many others in this study including LUCRC. LUCRC was proven to be critical for colorectal cancer cell growth, migration, invasion, and tumorigenesis. Other lncRNAs identified to be dysregulated in colorectal tumor tissues in this study might also be functional important in the development of colorectal cancer, which are worthy of future investigation.

The molecular mechanisms through which lncRNAs exert their functions are dependent on their cellular localization. In consistent with its cytosolic localization, LUCRC was found to affect the activation of UPR pathways and regulate the expression of UPR target genes, including BIP. Regarding how LUCRC affects UPR pathways, we propose that LUCRC might be able to form a RNP complex with sensory proteins such as ATF6α or IRE1α directly, and therefore regulating the activation of UPR. Alternatively, the cytoplasmic localization of LUCRC might make it a perfect candidate for miRNA sponge to regulate mRNA levels of key regulators in UPR pathway. Due to a small portion of LUCRC was found to be localized in the nucleus, we do not exclude the possibility that LUCRC could also function *in cis* to regulate the genes nearby, such as PKD1. Interestingly, PKD1 have been reported to be involved in UPR pathway (Tsutsuki et al., 2016; Wu et al., 2019). Thus, both the *in trans* and *in cis* models of LUCRC remain as interesting topics for future investigation.

When the cells are experiencing hypoxia and nutrient deficiency, the unfolded proteins in the ER will increase, which activates the UPR signaling pathway. Tumor cells are often in the microenvironment of endoplasmic reticulum stress, and they have evolved a protective mechanism that activates the UPR pathway to accelerate the folding of proteins in the cytoplasmic and clearance of unfolded proteins, thereby resisting the adverse effects of ER stress and promoting the survival of tumor cells. We propose that upregulation of LUCRC serves as one of such protective mechanisms in colorectal cancer, in which LUCRC activates UPR signaling pathway to promote colorectal tumor cell survival.

## Data Availability Statement

RNA-seq data were deposited in the Gene Expression Omnibus database under accession GSE136040.

## Ethics Statement

The studies involving human participants were reviewed and approved by Institutional Ethics Committee of Affiliated Nanhua Hospital, University of South China;Institutional Ethics Committee of The First Affiliated Hospital of Xiamen University. The patients/participants provided their written informed consent to participate in this study. The animal study was reviewed and approved by Xiamen Animal Care and Use Committee.

## Author Contributions

WL, X-SH, and FY conceived the original ideas, designed the project, and wrote the manuscript with inputs from G-HT, XC, J-CD, JD, X-TL, LX, and J-BL. G-HT and XC performed the majority of the experiments with participation from JD, X-TL, LX, and J-BL. J-CD performed all the bioinformatics analyses.

## Funding

This work was supported by National Natural Science Foundation of China (81761128015, 81861130370, 31871319, and 91953114), Fujian Province Health Education Joint Research Project (WKJ2016-2-09), Xiamen Science and Technology Project (2017S0091), Xiamen Science and Technology major projects (3502Z20171001-20170302), and the Fundamental Research Funds for the Central University (2013121036 and 20720190145) to WL. Hunan Province Clinical Medical Technology Innovation Guide Program to G-HT.

## Conflict of Interest

The authors declare that the research was conducted in the absence of any commercial or financial relationships that could be construed as a potential conflict of interest.
