# Supplementary material for: LncRNA LUCRC Regulates Colorectal Cancer Cell Growth and Tumorigenesis by Targeting Endoplasmic Reticulum Stress Response
Source: Front Genet. 2020 Jan 31;10:1409. doi: 10.3389/fgene.2019.01409 (PMC7005251; doi:10.3389/fgene.2019.01409)
Supplement: Supplementary file 1 [file DataSheet_1.pdf]

Supplementary Information

Supplementary Figure Legends

Figure S1. Gene ontology (GO), Kyoto encyclopedia of genes and genomes (KEGG) pathway and Hallmark analysis for genes dysregulated in colorectal tissue samples

(A, C, E) GO (A), KEGG (C) and Hallmark (E) analysis for genes up-regulated in colorectal tumor tissues as shown in Fig. 1D.

(B, D, F) GO (B), KEGG (D) and Hallmark (F) analysis for genes down-regulated in colorectal tumor tissues as shown in Fig. 1D.

Figure S2. Hundreds of lncRNAs were dysregulated in colorectal cancer

(A-D) UCSC genome browser views of RNA-seq as described in Fig. 1A for specific down-regulated lncRNAs were shown as indicated.

(E) The expression of lncRNAs as shown in Fig. 2E in a cohort of clinical colorectal tumor ($n = 647$) and normal ($n = 51$) samples from TCGA (The Cancer Genome Atlas).

(F) The knockdown efficiency of siRNAs targeting lncRNAs as described in Fig. 2F was examined by RT-qPCR analysis (± s.e.m., $^{**}P < 0.01$, $^{***}P < 0.001$).

Figure S3. LUCRC is a lncRNA with three exons and localized in the cytosol of cells

(A) UCSC genome browser view of RNA-seq as described in Fig. 1A for LUCRC was shown. The genomic location and the number of exons of LUCRC were depicted at the bottom.

(B) cDNA sequence of LUCRC (accession number: NR_135175.1).

(C, D) HCT116 cells were subjected to polysome profiling and the resultant fractions were subjected to RNA exaction and RT-qPCR analysis to examine the expression of LUCRC (C) and ACTIN (D). Fractions 1 to 4: free RNA (unbound RNA); Fractions 5 to 6: 40S; Fraction 7: 60S;

Fractions 8 to 10: monosome; Fractions 11 to 20: polysome.

(E) HCT116 cells were subjected to cellular fractionation followed by RNA extraction and RT-qPCR analysis to quantify the amount of mRNA as indicated in both nucleus and cytosol of the cells. ACTIN and MALAT1 served as markers for cytosolic and nuclear fraction, respectively.

(F, G) RKO (F) and DLD1 (G) cells were transfected with control siRNA (siCTL) or siRNA specifically targeting LUCRC (siLUCRC) for duration as indicated followed by cell proliferation assay (± s.e.m., $*P < 0.05$, $**P < 0.01$).

(H) The expression of LUCRC in a cohort of clinical colorectal tumor samples at different stages (stage I, n=111; stage II, n=238; stage III, n=183; stage IV, n=90) and normal samples (n=51) from TCGA.

(I) Kaplan-Meier survival analyses for OS (overall survival) (n = 361) of colorectal cancer patients using LUCRC as input.

Figure S4. LUCRC was required for the expression of genes involved in ER stress response, including BIP

(A) Correlation of the effects of LUCRC on whole transcriptome based on RNA-seq between two biological repeats.

(B) MA plot shows the fold change (FC, siCTL/siLUCRC, log2) against the average of normalized counts for samples as described in Fig. 4A. Red dots represented genes with significant change in response to LUCRC knockdown ($q <0.05$), and blue line indicated fold change of 1.5.

(C) The expression of BIP in a cohort of clinical colorectal tumor samples at different stages (stage I, n=111; stage II, n=238; stage III, n=183; stage IV, n=90) and normal samples (n=51) from TCGA.

(D) Kaplan-Meier survival analyses for OS (overall survival) (n = 361) of colorectal cancer patients using BIP as input.

Supplementary Table Legend

Table S1. Sequence information for all qPCR primers used in this study. Sequence information of qPCR primers designed to detect the expression of *MYC*, *CCND1*, *FAM83H-AS1*, *MNX1-AS1*, *RNASEH1-AS1*, *LOC105370333*, *OLMALINC*, *SNHG8*, *LUCRC*, *VPS9D1-AS1*, *LOC101927811*, *GAS5*, *MALAT1* and *BIP*. F: forward; R: reverse.

Table S2. Targeting sequence for all siRNAs used in this study. Targeting sequences of siRNAs designed to knock down *FAM83H-AS1*, *MNX1-AS1*, *RNASEH1-AS1*, *LOC105370333*, *OLMALINC*, *SNHG8*, *LUCRC*, *VPS9D1-AS1*, *LOC101927811* or *GAS5* were shown.

Table S3. Genes regulated by LUCRC in HCT116 cells as detected by RNA-seq analysis. HCT116 cells transfected with control siRNA (siCTL) and siRNA specifically targeting LUCRC (siLUCRC) for three days were subjected to RNA-seq analysis, and two biological repeats were analyzed. Genes positively- and negatively-regulated by LUCRC were highlighted in red and blue, respectively (Fold change (FC) > 1.5, q<0.05).

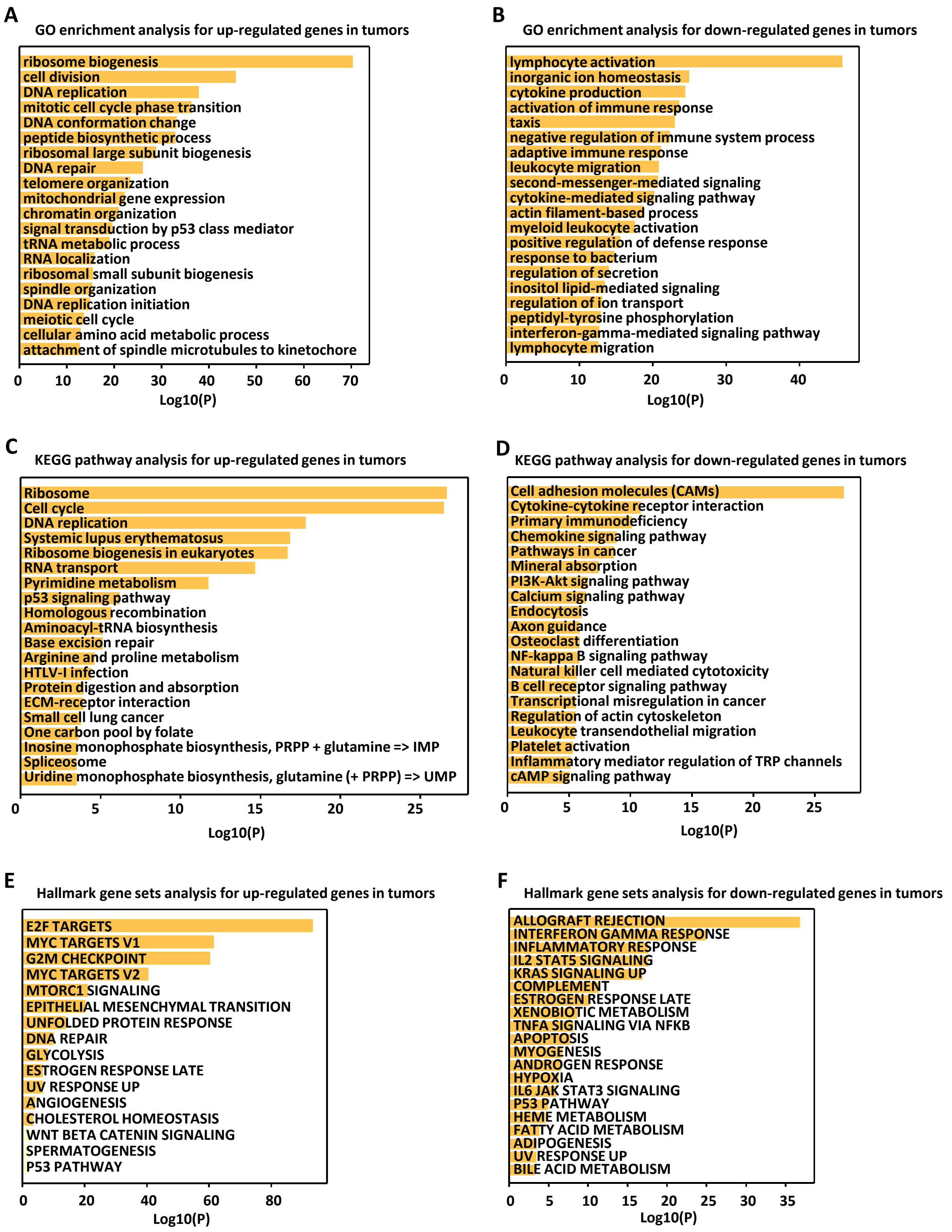
A
GO enrichment analysis for up-regulated genes in tumors
ribosome biogenesis
cell division
DNA replication
mitotic cell cycle phase transition
DNA conformation change
peptide biosynthetic process
ribosomal large subunit biogenesis
DNA repair
telomere organization
mitochondrial gene expression
chromatin organization
signal transduction by p53 class mediator
tRNA metabolic process
RNA localization
ribosomal small subunit biogenesis
spindle organization
DNA replication initiation
meiotic cell cycle
cellular amino acid metabolic process
attachment of spindle microtubules to kinetochore
0 10 20 30 40 50 60 70
Log10(P)
B
GO enrichment analysis for down-regulated genes in tumors
lymphocyte activation
inorganic ion homeostasis
cytokine production
activation of immune response
taxis
negative regulation of immune system process
adaptive immune response
leukocyte migration
second-messenger-mediated signaling
cytokine-mediated signaling pathway
actin filament-based process
myeloid leukocyte activation
positive regulation of defense response
response to bacterium
regulation of secretion
inositol lipid-mediated signaling
regulation of ion transport
peptidyl-tyrosine phosphorylation
interferon-gamma-mediated signaling pathway
lymphocyte migration
0 10 20 30 40
Log10(P)
C
KEGG pathway analysis for up-regulated genes in tumors
Ribosome
Cell cycle
DNA replication
Systemic lupus erythematosus
Ribosome biogenesis in eukaryotes
RNA transport
Pyrimidine metabolism
p53 signaling pathway
Homologous recombination
Aminoacyl-tRNA biosynthesis
Base excision repair
Arginine and proline metabolism
HTLV-I infection
Protein digestion and absorption
ECM-receptor interaction
Small cell lung cancer
One carbon pool by folate
Inosine monophosphate biosynthesis, PRPP + glutamine => IMP
Spliceosome
Uridine monophosphate biosynthesis, glutamine (+ PRPP) => UMP
0 5 10 15 20 25
Log10(P)
D
KEGG pathway analysis for down-regulated genes in tumors
Cell adhesion molecules (CAMs)
Cytokine-cytokine receptor interaction
Primary immunodeficiency
Chemokine signaling pathway
Pathways in cancer
Mineral absorption
PI3K-Akt signaling pathway
Calcium signaling pathway
Endocytosis
Axon guidance
Osteoclast differentiation
NF-kappa B signaling pathway
Natural killer cell mediated cytotoxicity
B cell receptor signaling pathway
Transcriptional misregulation in cancer
Regulation of actin cytoskeleton
Leukocyte transendothelial migration
Platelet activation
Inflammatory mediator regulation of TRP channels
cAMP signaling pathway
0 5 10 15 20 25
Log10(P)
E
Hallmark gene sets analysis for up-regulated genes in tumors
E2F TARGETS
MYC TARGETS V1
G2M CHECKPOINT
MYC TARGETS V2
MTORC1 SIGNALING
EPITHELIAL MESENCHYMAL TRANSITION
UNFOLDED PROTEIN RESPONSE
DNA REPAIR
GLYCOLYSIS
ESTROGEN RESPONSE LATE
UV RESPONSE UP
ANGIOGENESIS
CHOLESTEROL HOMEOSTASIS
WNT BETA CATENIN SIGNALING
SPERMATOGENESIS
P53 PATHWAY
0 20 40 60 80
Log10(P)
F
Hallmark gene sets analysis for down-regulated genes in tumors
ALLOGRAFT REJECTION
INTERFERON GAMMA RESPONSE
INFLAMMATORY RESPONSE
IL2 STAT5 SIGNALING
KRAS SIGNALING UP
COMPLEMENT
ESTROGEN RESPONSE LATE
XENOBIOTIC METABOLISM
TNFA SIGNALING VIA NFKB
APOPTOSIS
MYOGENESIS
ANDROGEN RESPONSE
HYPOXIA
IL6 JAK STAT3 SIGNALING
P53 PATHWAY
HEME METABOLISM
FATTY ACID METABOLISM
ADIPOGENESIS
UV RESPONSE UP
BILE ACID METABOLISM
0 5 10 15 20 25 30 35
Log10(P)

Figure S1

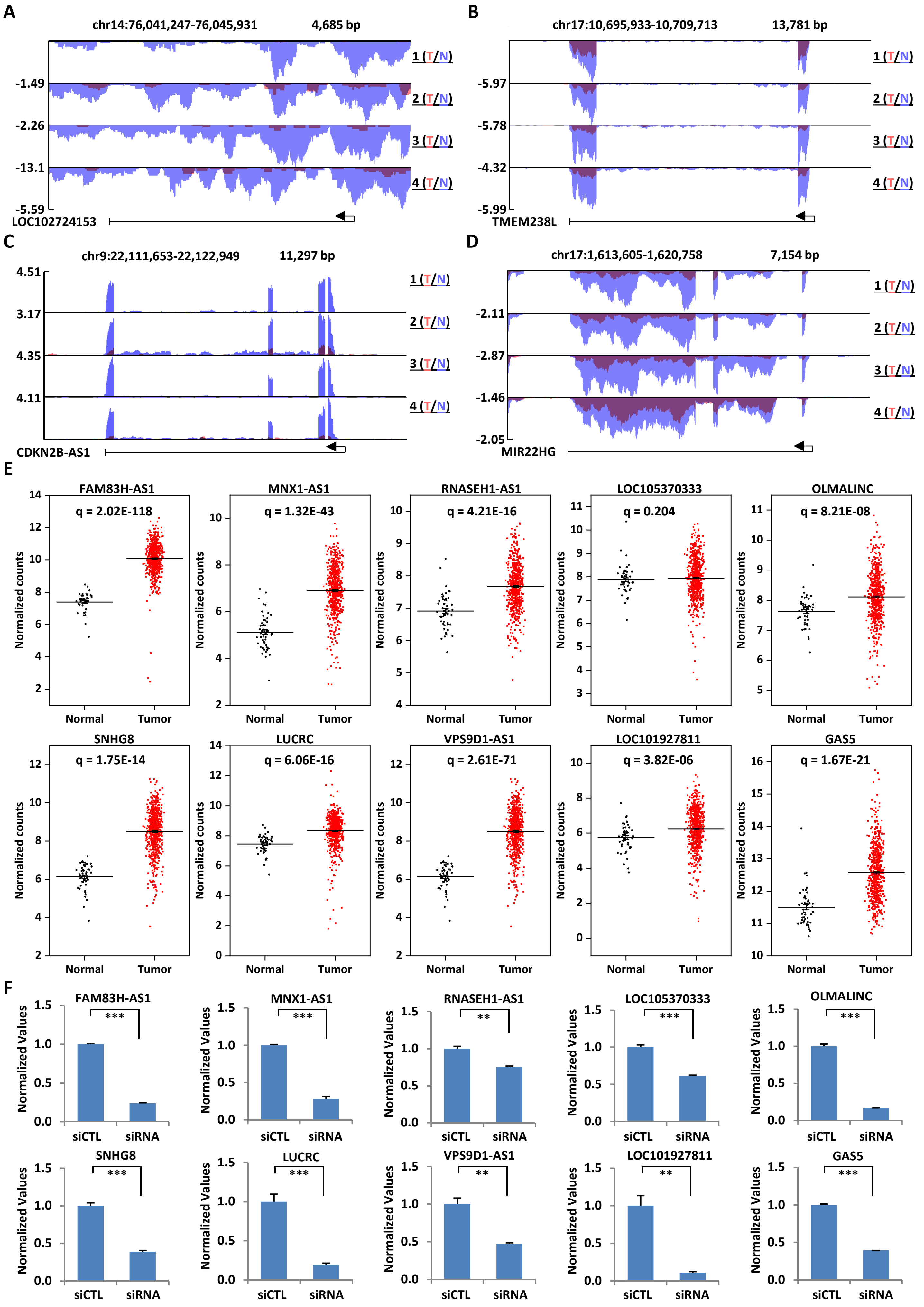
A
chr14:76,041,247-76,045,931 4,685 bp
-1.49
-2.26
-13.1
-5.59
1 (T/N)
2 (T/N)
3 (T/N)
4 (T/N)
LOC102724153
B
chr17:10,695,933-10,709,713 13,781 bp
-5.97
-5.78
-4.32
-5.99
TMEM238L
C
chr9:22,111,653-22,122,949 11,297 bp
4.51
3.17
4.35
4.11
CDKN2B-AS1
D
chr17:1,613,605-1,620,758 7,154 bp
-2.11
-2.87
-1.46
-2.05
MIR22HG
E
FAM83H-AS1 q = 2.02E-118
MNX1-AS1 q = 1.32E-43
RNASEH1-AS1 q = 4.21E-16
LOC105370333 q = 0.204
OLMALINC q = 8.21E-08
SNHG8 q = 1.75E-14
LUCRC q = 6.06E-16
VPS9D1-AS1 q = 2.61E-71
LOC101927811 q = 3.82E-06
GAS5 q = 1.67E-21
Normalized counts
Normal
Tumor
F
FAM83H-AS1 ***
MNX1-AS1 ***
RNASEH1-AS1 **
LOC105370333 ***
OLMALINC ***
SNHG8 ***
LUCRC ***
VPS9D1-AS1 **
LOC101927811 **
GAS5 ***
Normalized Values
siCTL
siRNA

Figure S2

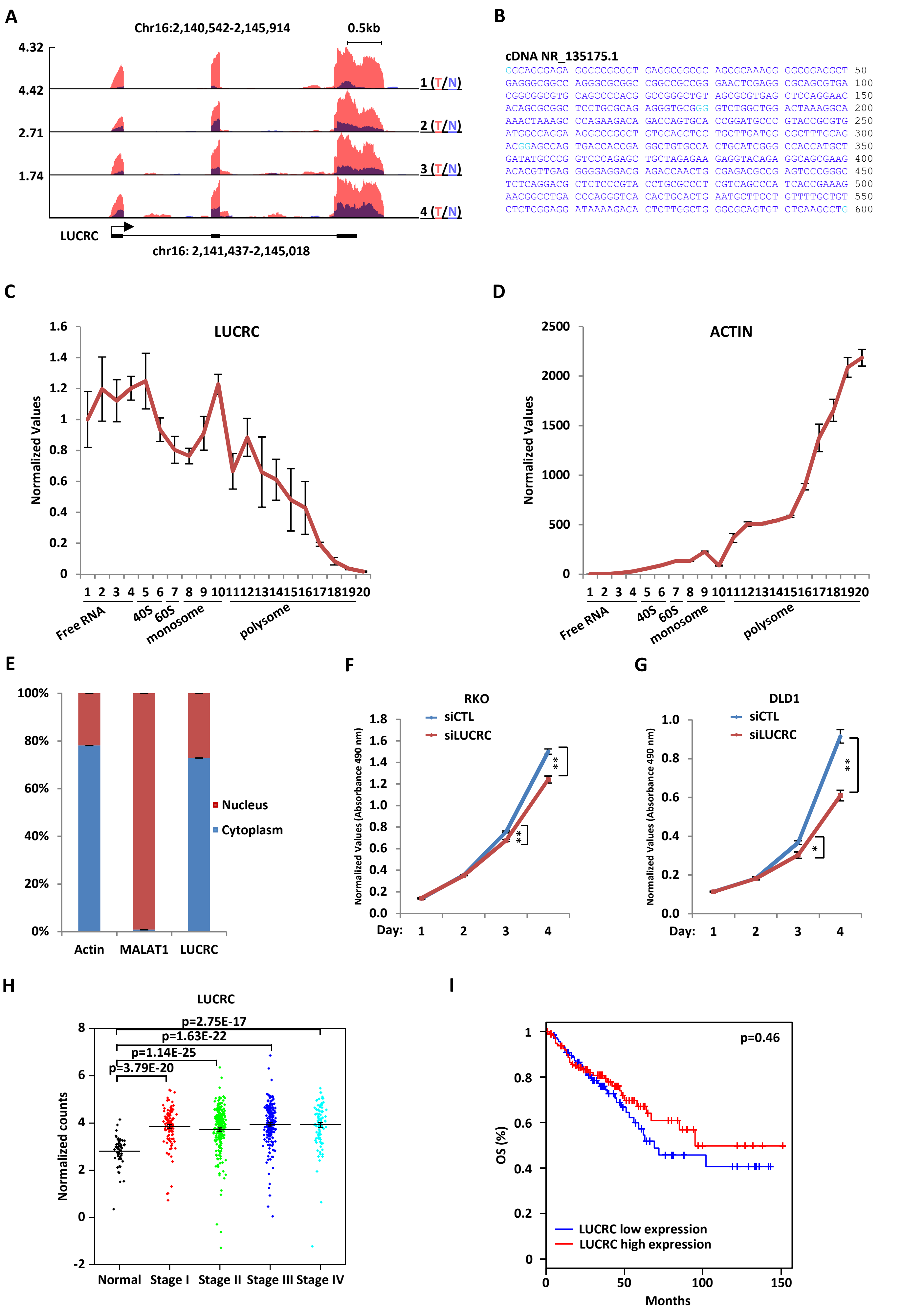
A
Chr16:2,140,542-2,145,914
0.5kb
4.32
4.42
2.71
1.74
1 (T/N)
2 (T/N)
3 (T/N)
4 (T/N)
LUCRC
chr16: 2,141,437-2,145,018
B
cDNA NR_135175.1
GGCAGCGAGA GGCCCGCGCT GAGGCGGCGC AGCGCAAAGG GGCGGACGCT 50
GAGGGCGGCC AGGGCGCGGC CGGCCGCCGG GAACTCGAGG CGCAGCGTGA 100
CGGCGGCGTG CAGCCCCACG GCCGGGCTGT AGCGCGTGAG CTCCAGGAAC 150
ACAGCGCGGC TCCTGCGCAG AGGGTGCGGG GTCTGGCTGG ACTAAAGGCA 200
AAACTAAAGC CCAGAAGACA GACCAGTGCA CCGGATGCCC GTACCGCGTG 250
ATGGCCAGGA AGGCCCGGCT GTGCAGCTCC TGCTTGATGG CGCTTTGCAG 300
ACGGAGCCAG TGACCACCGA GGCTGTGCCA CTGCATCGGG CCACCATGCT 350
GATATGCCCG GTCCCAGAGC TGCTAGAGAA GAGGTACAGA GGCAGCGAAG 400
ACACGTTGAG GGGGAGGACG AGACCAACTG CGAGACGCCG AGTCCCGGGC 450
TCTCAGGACG CTCTCCCGTA CCTGCGCCCT CGTCAGCCCA TCACCGAAAG 500
AACGGCCTGA CCCAGGGTCA CACTGCACTG AATGCTTCCT GTTTTGCTGT 550
CTCTCGGAGG ATAAAAGACA CTCTTGGCTG GGCGCAGTGT CTCAAGCCTG 600
C
LUCRC
Normalized Values
1.6
1.4
1.2
1
0.8
0.6
0.4
0.2
0
1 2 3 4 5 6 7 8 9 10 11 12 13 14 15 16 17 18 19 20
Free RNA
40S
60S
monosome
polysome
D
ACTIN
Normalized Values
2500
2000
1500
1000
500
0
1 2 3 4 5 6 7 8 9 10 11 12 13 14 15 16 17 18 19 20
Free RNA
40S
60S
monosome
polysome
E
100%
80%
60%
40%
20%
0%
Nucleus
Cytoplasm
Actin
MALAT1
LUCRC
F
RKO
siCTL
siLUCRC
Normalized Values (Absorbance 490 nm)
1.8
1.6
1.4
1.2
1.0
0.8
0.6
0.4
0.2
0.0
Day: 1 2 3 4
**
**
G
DLD1
siCTL
siLUCRC
Normalized Values (Absorbance 490 nm)
1.0
0.8
0.6
0.4
0.2
0.0
Day: 1 2 3 4
**
*
H
LUCRC
p=2.75E-17
p=1.63E-22
p=1.14E-25
p=3.79E-20
Normalized counts
8
6
4
2
0
-2
Normal
Stage I
Stage II
Stage III
Stage IV
I
p=0.46
OS (%)
1
0.8
0.6
0.4
0.2
0
0 50 100 150
Months
LUCRC low expression
LUCRC high expression

Figure S3

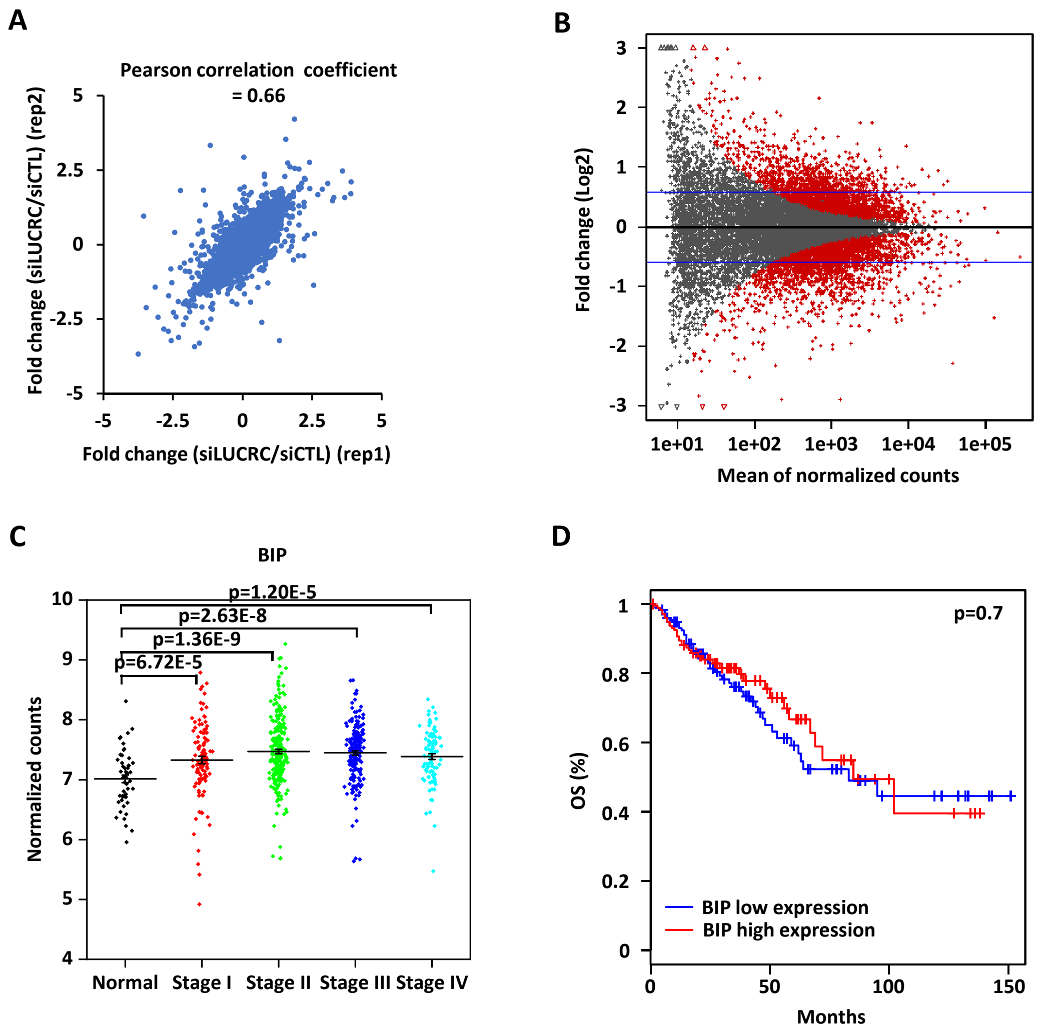
A
Pearson correlation coefficient
= 0.66
Fold change (siLUCRC/siCTL) (rep2)
Fold change (siLUCRC/siCTL) (rep1)
5
2.5
0
-2.5
-5
B
Fold change (Log2)
Mean of normalized counts
1e+01
1e+02
1e+03
1e+04
1e+05
C
BIP
p=1.20E-5
p=2.63E-8
p=1.36E-9
p=6.72E-5
Normalized counts
Normal
Stage I
Stage II
Stage III
Stage IV
D
p=0.7
OS (%)
Months
BIP low expression
BIP high expression

Figure S4
